# Symptoms Six Weeks After COVID-19 Are Reduced Among US Health Care Personnel Receiving Additional Vaccine Doses During the Omicron Period, December 2021–April 2022

**DOI:** 10.1093/ofid/ofae545

**Published:** 2024-09-25

**Authors:** Nicholas M Mohr, Ian D Plumb, Eliezer Santos León, Malea Pinckney, Karisa K Harland, Anusha Krishnadasan, Karin F Hoth, Fernand Rwamwejo, John P Haran, Melissa Briggs-Hagen, Eric Kontowicz, David A Talan, for the Project PREVENT Network, Sydney Krispin, Allison Schuette, Jillian Tozloski, Lili O’Brian, Laura McCormick, Karen Hopcia, Theresa M Orechia, Alexander B Hill, Gabrielle Donohoe, Lily R Johnsky, Jordyn M Fofi, Steven E Miyawaki, Jenson J Kaithamattam, Michelle Chung, Nikita A Umale, Mohammad Adrian Hasdianda, Guruprasad Jambaulikar, Tala Teymour, Maria Davila, Suzette Fernandez, Elizabeth Krebs, Joshua Tiao, Stacey Wisniewski, Gaynell Bernadas-Hunt, Alexander Vandeerlin, Domnic Bett, Cathryn Leggio, Alexandria Henderson, Reynaldo Padilla, Cynthia Delgado, Madeleine Manahan, Susana Hacopian, Vincent E Yu, Melanie Potts, Jessica Kuo, Alyssa Fowlds, Lidia Choxom, Amy Dakos, Denise Tritt, Zoe Speight, Laurie Kemble, Danielle Beckham, Cecile Hermanns, Geneatra Green, Rachel Marrs, Katherine Schneider, Catherine Fairfield, Shannon Landers, Eliezer Santos, Nathan R Kramer, Fred Ullrich, Virginia Mangolds, Morgan Nelson, Abigail Lopes, James Galbraith, Lucia Solis, Rebekah Peacock, Alan Jones, Bhagyashri Navalkele, Savannah Vann, Alexander Kile, Joel Rodgers, Nivedita Patkar, Delissa Tidwell-Hand, Whitney Covington, Michael C Kurz, Peter Poerzgen, Megan Fuentes, Layla A Anderson, Kyle A Steinbock, Alison Zelikoff, John B Lynch, Jennifer Smith, Glen R Abedi, Sankan Nyanseor, Christopher K Watts, Jade James Gist, Sharon Saydah, Matthew McCullough, Elizabeth Krebs, Howard A Smithline, Peter C Hou, Lilly C Lee, Stephen C Lim, Gregory J Moran, Mark T Steele, David G Beiser, Brett Faine, Utsav Nandi, Walter A Schrading, Brian Chinnock, Anne Chipman, Christine D Crider, Frank LoVecchio, Alysia Horcher, Kelli Wallace, Lisandra Uribe, Kavitha Pathmarajah, Abigail L Girardin, Dean M Hashimoto, Monica Bahamon, Michelle St. Romain, Efrat Kean, Zachary Mankoff, Amy Stubbs, Sara Roy, Gregory Volturo, Amanda Higgins, James Galbraith, James C Crosby, Mary Mulrow, Eva Gonzalez, J Priyanka Vakkalanka

**Affiliations:** University of Iowa, City Iowa, Iowa, USA; Coronavirus and Other Respiratory Viruses Division, National Center for Immunization and Respiratory Diseases, Atlanta, Georgia, USA; University of Iowa, City Iowa, Iowa, USA; Iowa State University, Ames, Iowa, USA; University of Iowa, City Iowa, Iowa, USA; Olive View-and Ronald Reagan University of California Los Angeles Medical Center, Los Angeles, California, USA; University of Iowa, City Iowa, Iowa, USA; Thomas Jefferson University Hospital, Philadelphia, Pennsylvania, USA; University of Massachusetts Chan Medical School, Worcester, Massachusetts, USA; Coronavirus and Other Respiratory Viruses Division, National Center for Immunization and Respiratory Diseases, Atlanta, Georgia, USA; University of Iowa, City Iowa, Iowa, USA; University of Iowa, City Iowa, Iowa, USA; Olive View-and Ronald Reagan University of California Los Angeles Medical Center, Los Angeles, California, USA

**Keywords:** COVID-19, post-acute COVID-19 syndrome, COVID-19 vaccines, vaccine efficacy

## Abstract

**Background:**

The objective of this study was to test the hypothesis that subsequent doses of the coronavirus disease 2019 (COVID-19) vaccine are associated with lower incidence of COVID-19-like symptoms at 6 weeks after infection.

**Methods:**

This study was a case–control analysis of health care personnel in an ongoing multicenter COVID-19 vaccine effectiveness study. We enrolled participants at the time of COVID-19-like symptoms between December 19, 2021, and April 27, 2022, which corresponded to the early Omicron-predominant period after original monovalent severe acute respiratory syndrome coronavirus 2 additional vaccination doses became available. Our outcome was self-reported symptoms completed 6 weeks after the onset of symptoms.

**Results:**

We enrolled 2478 participants, of whom 1422 (57%) had COVID-19. The prevalence of symptoms at 6 weeks was 26% (n = 373) in those with COVID-19 and 18% (n = 195) in those without COVID-19. Fatigue (11%) and difficulty sleeping (7%) were most strongly associated with COVID-19. A total of 1643 (66%) participants received a subsequent vaccine dose (after the primary series). Participants with COVID-19 who had received a subsequent vaccination had lower odds of symptoms at 6 weeks (adjusted odds ratio [aOR], 0.55; 95% CI, 0.43–0.70), but this relationship was not observed in those without COVID-19 (aOR, 0.87; 95% CI, 0.59–1.29).

**Conclusions:**

Health care personnel who received subsequent doses of original monovalent COVID-19 vaccine had a lower prevalence of symptoms at 6 weeks than those that did not.

Coronavirus disease 2019 (COVID-19) has caused >1 million reported deaths in the United States and has put significant strain on health care systems [1–3]. Following COVID-19, an estimated 6%–10% of survivors at 3 months experience prolonged symptoms including neurological, respiratory, and cardiovascular symptoms [4, 5]. These symptoms can lead to disability and reduced quality of life. Prolonged symptoms can be especially consequential among health care personnel (HCP) due to the potential adverse impact on health care system capacity [6–9].

Prolonged symptoms are most prevalent in those with more severe COVID-19, though they may occur with mild disease [6]. Initial COVID-19 vaccination has been associated with a lower risk of prolonged symptoms in individuals with subsequent COVID-19 [10, 11], but the role of vaccine doses after the primary series is less clear [9, 12, 13]. Because of post-COVID symptoms' impact on daily functioning and work productivity, vaccinations or pharmacotherapy that can limit prolonged symptoms—even beyond their role in preventing severe disease—would be valuable [9, 14].

In our prior study, we concluded that receipt of the initial COVID-19 vaccine series was associated with decreased prevalence of symptoms 6 weeks after infection in HCP infected with the early severe acute respiratory syndrome coronavirus 2 (SARS-CoV-2) strains, but we did not consider the role of subsequent vaccine doses or include infections during the Omicron-predominant period [11]. The objective of this study was to measure the association between receipt of an original monovalent mRNA COVID-19 additional vaccine dose before infection and the prevalence of 6-week symptoms among HCP with COVID-19. To assess the robustness of our findings, we also evaluated the extent to which 6-week symptoms were associated with SARS-CoV-2 detection. We hypothesized that 6-week symptoms would be less prevalent among HCP who had received an additional vaccine dose compared with other HCP, among participants with COVID-19. These aims were tested in the context of an ongoing vaccine effectiveness study during the Omicron-predominant period of the COVID-19 pandemic.

## METHODS

### Study Design, Data Collection, and Population

This study was a subanalysis of a case–control study in the multicenter Preventing Emerging Infections through Vaccine Effectiveness Testing Project (Project PREVENT), including both those with COVID-19-like illness who tested positive (cases) and negative (controls) for SARS-CoV-2. Project PREVENT is a 16-site study of HCP who developed symptoms of COVID-19 and were tested for SARS-CoV-2. We included those tested between December 19, 2021, and April 27, 2022, which corresponded to the early Omicron-predominant period. During this period, original monovalent COVID-19 additional vaccination doses (after the initial 2-dose series) were available. Individuals with prior SARS-CoV-2-positive nasal, nasopharyngeal, or oral tests were not eligible for participation. Participants provided informed consent and completed standardized electronic surveys. All vaccination and SARS-CoV-2 tests were validated by local site teams using source document verification. The methods of Project PREVENT have been reported previously [15]. This report was prepared in accordance with the Strengthening the Reporting of Observational Studies in Epidemiology (STROBE) guidelines [16]. This activity was reviewed by the Centers for Disease Control and Prevention (CDC) and was conducted consistent with applicable federal law and CDC policy [17]. At 15 of the 16 participating sites, this research was deemed nonresearch public health assessment. The remaining site's institutional review board (IRB) considered this project to be human subject research, and the local IRB approved the project. Written consent was obtained from all participants.

All participants reported COVID-19-like symptoms within 14 days of a qualifying test and were stratified into those with a positive antigen or polymerase chain reaction (PCR) test result for SARS-CoV-2 and those with a negative SARS-CoV-2 test result by PCR. Participants with a negative antigen test only were not eligible because of the low sensitivity of the antigen test. False-positive test results were assumed to be low due to previous validation studies of PCR, immunoassays, and rapid antigen tests [18–20]. We defined COVID-19-like symptoms to be the presence of abdominal pain, bruised toes or feet, changes in ability to taste or smell, chest pain or tightness, chills, cough, diarrhea, fatigue, fever, headache, loss of appetite, myalgia, nausea or vomiting, rhinorrhea, rigors, sever respiratory illness including pneumonia, shortness of breath or difficulty breathing, sinus or nasal congestions, and sore throat. Symptom severity was not assessed at enrollment. Participants provided baseline information at the time of enrollment (during the 60 days after symptom onset) and received a follow-up survey 6 weeks after symptom onset (the baseline survey prompted for symptoms at baseline, and the 6-week survey prompted for symptoms at 6 weeks). Participants included in this analysis had received a primary mRNA COVID-19 vaccination series. The primary exposure was receipt of a COVID-19 additional vaccine dose after the primary series at least 14 days before the onset of qualifying symptoms. Participants were excluded from this analysis if they never received a primary mRNA vaccination series, if they received a non-mRNA COVID-19 vaccine, if they received a COVID-19 additional vaccine dose <14 days before the onset of qualifying COVID-19 symptoms, or if the 6-week follow-up survey was not completed during the 10 weeks after symptom onset.

### Definitions

Prolonged symptoms were defined as at least 1 of the following: fatigue, cough, shortness of breath, congestion, chest pain, headache, dizziness, joint pain, muscle weakness, movement problems (such as tremor), sore throat, loss of taste or smell, diarrhea, nausea, vomiting, abdominal pain, confusion, memory difficulty, concentration difficulty, fever, chills, weight loss, weight gain, exercise difficulty, sleep difficulty, anxiety or panic, and depression [21]. On the 6-week survey, we collected information about the presence of symptoms and their self-rated severity (mild, moderate, and severe). We subsequently categorized 6-week symptoms into categories based on symptom groups from prior studies: general symptoms, respiratory symptoms, cardiac symptoms, neurologic symptoms, gastrointestinal symptoms, psychiatric symptoms, and any other reported symptoms (Supplementary Table 1) [11].

We collected information about underlying health conditions both from self-report and from medical records abstracted by local project teams during any COVID-19-related visits within 14 days of the index symptoms.

### Outcomes

Our primary outcome was the prevalence of any symptoms of at least moderate severity (according to self-report) 6 weeks after illness onset. We stratified the analysis by SARS-CoV-2 infection status (ie, positive vs negative) at the time of enrollment and compared the outcome within each group by vaccination status (ie, primary series and no COVID-19 vaccine additional doses vs primary series and at least 1 additional dose). We also conducted a subgroup analysis including only those who had received an additional vaccine dose within 16 weeks of symptom onset (compared with all those who had not received any additional doses) because vaccine effectiveness data suggest that this is the period of greatest vaccine efficacy [22–24].

### Statistical Analysis

We used multivariable logistic regression to model the odds of reporting symptoms at 6 weeks, with an additional vaccine dose as the primary exposure variable. We conducted this analysis separately among those with confirmed COVID-19 and those with COVID-19-like illness but who tested negative for SARS-CoV-2. In the multivariable model, we selected the following covariates a priori: age group, race and ethnicity, days to follow-up (as some participants completed their survey after 6 weeks), job classification, and the presence of at least 2 comorbidities. All covariates were modeled as fixed effects, and we did not include random effects to account for sites as the mixed-effects model was comparable to a fixed-effects model. Comorbidities were constructed as a dichotomous variable to represent 2 or more comorbidities reported at baseline as an indicator of presence of a chronic illness, from the following list: asthma, allergic rhinitis, chronic obstructive pulmonary disease/emphysema, other chronic lung disease, hypertension, coronary artery disease, other heart condition, stroke, type I diabetes, type II diabetes, unspecified diabetes type, chronic kidney disease, dialysis, solid organ transplant, hematopoietic stem cell transplant, autoimmune or rheumatologic disease, other immunosuppressing condition, active cancer, deep vein thrombosis or pulmonary embolism, chronic liver disease, depression or other mood disorder, anxiety/obsessive-compulsive/trauma- or stressor-related disorder, other mental health condition, movement or motor disorders, cognitive/neurodevelopmental disorder, alcohol use disorder, sleep disorder, other medical conditions, obesity/overweight, pregnant, current/former smoker. An additional sensitivity analysis was conducted to compare those who received a vaccine within 16 weeks before infection with those whose latest vaccine was >16 weeks before infection using similar multivariable modeling methods.

We performed a secondary analysis to model the odds of reporting persistent symptoms (including only 6-week symptoms that were also reported at baseline), with an additional vaccine dose as the primary exposure variable using the multivariable modeling method described above.

We used a chart to summarize the prevalence of individual symptoms in those with and without COVID-19 infection. Additionally, we compared the association between vaccination status and 6-week symptoms in both the COVID-19 and non-COVID-19 groups using prevalence, difference in proportions tests, and odds ratios with their corresponding 95% confidence intervals. For analysis purposes, we considered proportions to be different if the 95% confidence interval of the difference did not include the null value (0).

## RESULTS

### Participant Characteristics

**Figure 1.**
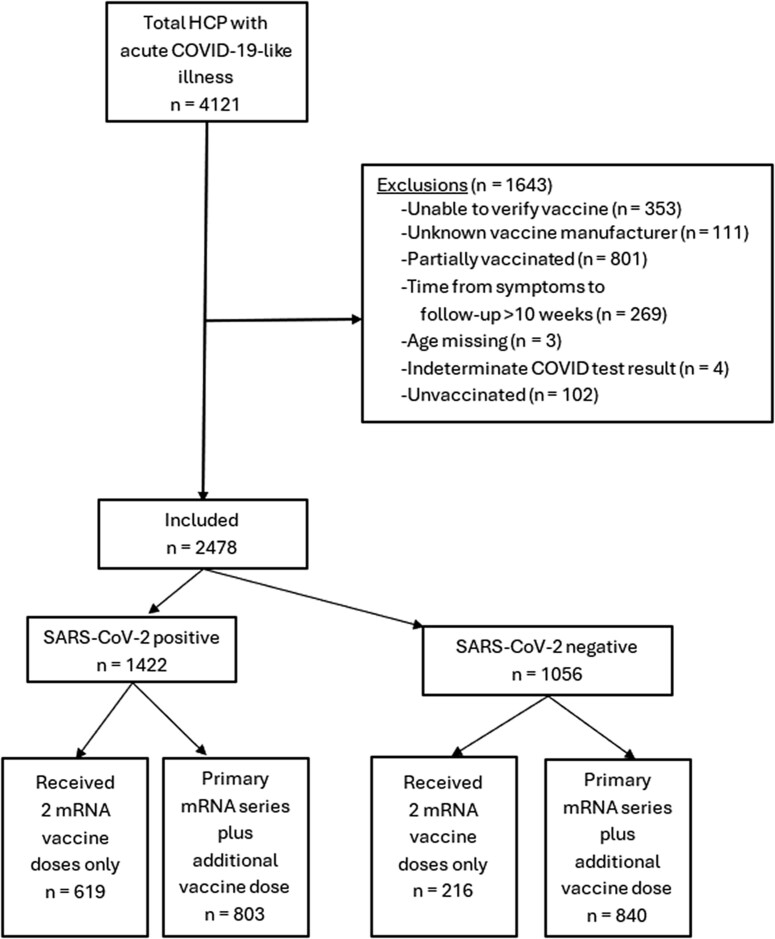
Flow diagram of US health care personnel, December 2021 to April 2022. Abbreviations: COVID-19, coronavirus disease 2019; HCP, health care personnel; SARS-CoV-2, severe acute respiratory syndrome coronavirus 2.

Between December 19, 2021, and April 27, 2022, 4121 HCP with acute illness were enrolled into PREVENT, and 2478 (60.1%) HCP were included in this study. We excluded HCP who were not vaccinated (n = 102, 2.5%) or were partially vaccinated (801, 19.4%), those for whom vaccination could not be verified (353, 8.6%), those for whom the vaccine manufacturer could not be determined (111, 2.7%), those who did not complete their follow-up survey within 10 weeks of symptom onset (269, 6.5%), those who had an indeterminant COVID-19 test result (4, 0.1%), and those who did not report their age (3, 0.1%) (Figure 1). Among included HCP, baseline surveys were completed a median (interquartile range [IQR]) of 3.0 (2.3–4.0) weeks after symptom onset, and 6-week surveys were completed at a median (IQR) of 6.0 (6.0–6.3) weeks after symptom onset. Most participants (77.6%, n = 1924) worked in acute care hospitals, and 1136 (45.8%) were physicians or nurses. Laboratory-confirmed COVID-19 occurred in 1422 (57.4%) participants (Figure 1). SARS-CoV-2-positive participants had either fever or cough as an initial symptom in 71.9% of cases (n = 1022), and this was higher than in those with negative SARS-CoV-2 tests (n = 472, 44.7%; difference, 27.2 percentage points [PPs]; 95% CI, 23.3–31.1 PPs). Among 1422 participants with COVID-19, the most frequently reported symptoms during acute illness were fatigue (68.4%), congestion (67.9%), and headache (65.4%). Among the 1056 SARS-CoV-2-negative participants, the most frequently reported symptoms were headache (54.1%), congestion (53.7%), and sore throat (53.0%). No participants were hospitalized for acute COVID-19, and no participants died.

A total of 1643 (66.3%) participants had received a subsequent COVID-19 vaccine dose (Figure 1), with a higher proportion of controls (n = 840, 79.5%) receiving a subsequent dose compared with cases (n = 803, 56.5%; difference, 25.3 PPs; 95% CI, 21.3–29.2 PPs). Receipt of a COVID-19 additional vaccine dose was more likely to be reported among physicians (cases: 11.3 PPs; 95% CI, 8.0–14.6 PPs; controls: 6.9 PPs; 95% CI, 2.5–11.3 PPs), those with a graduate or professional degree (cases: 19.0 PPs; 95% CI, 13.9–24.0 PPs; controls: 18.9 PPs; 95% CI, 11.7–26.0 PPs), and those who were non-Hispanic White (cases: 5.5 PPs; 95% CI, 1.0–9.9 PPs; controls: 7.7 PPs; 95% CI, 1.0–14.4 PPs) in both the case and control populations (Table 1). Most participants received the Pfizer-BioNTech vaccine for their primary series (n = 1837, 74.1%) and for the additional dose (n = 1281, 51.7%). Only 86 (3.5%) received a heterologous vaccine product (Table 1). Among those who had COVID-19, fever or cough as the initial symptom was lower in those who had received the additional vaccine dose than in those who had not (63.9% vs 82.2%; difference, 18.3 PPs; 95% CI, 13.7–23.0 PPs) (Supplementary Table 2).

**Table 1.** US Health Care Personnel Participant Characteristics During the Omicron-Predominant Period by SARS-CoV-2 Vaccination Status, December 2021 and April 2022

	SARS-CoV-2 Positive n = 1422			SARS-CoV-2 Negative n = 1056		
	Primary Series Only n = 619	Primary Series Plus Additional Vaccine Dose n = 803	Percentage Point Difference (95% CI)	Primary Series Only n = 216	Primary Series Plus Additional Vaccine Dose n = 840	Percentage Point Difference (95% CI)
Age						
18–29 y	161 (26.0)	161 (20.0)	6.0 (1.4 to 10.5)	57 (26.4)	155 (18.5)	7.9 (1.2 to 14.7)
30–39 y	183 (29.6)	286 (35.6)	−6.1 (−11.1 to −1.0)	87 (40.3)	297 (35.4)	4.9 (−2.7 to 12.5)
40–49 y	137 (22.1)	164 (20.4)	1.7 (−2.7 to 6.1)	37 (17.1)	164 (19.5)	−2.4 (−8.4 to 3.6)
50–64 y	133 (21.5)	175 (21.8)	−0.3 (−4.8 to 4.2)	34 (15.7)	208 (24.8)	−9.0 (−15.0 to −3.1)
≥65 y	5 (0.8)	17 (2.1)	−1.3 (−2.7 to 0.1)	1 (0.5)	16 (1.9)	−1.4 (−3.0 to 0.1)
Sex						
Male	96 (15.5)	140 (17.4)	−1.9 (−5.9 to 2.1)	27 (12.5)	152 (18.1)	−5.6 (−11.0 to −0.2)
Female	520 (84.0)	663 (82.6)	1.4 (−2.6 to 5.5)	188 (87.0)	687 (81.8)	5.3 (−0.2 to 10.7)
Missing	3 (0.5)	0	0.5 (−0.2 to 1.2)	1 (0.5)	1 (0.1)	0.3 (−0.9 to 1.6)
Race and ethnic group						
White, non-Hispanic	468 (75.6)	651 (81.1)	−5.5 (−9.9 to −1.0)	160 (74.1)	687 (81.8)	−7.7 (−14.4 to −1.0)
Black, non-Hispanic	76 (12.3)	42 (5.2)	7.0 (3.9 to 10.2)	19 (8.8)	26 (3.1)	5.7 (1.5 to 9.9)
Hispanic or Latino	54 (8.7)	53 (6.6)	2.1 (−0.8 to 5.1)	23 (10.6)	53 (6.3)	4.3 (−0.4 to 9.1)
Other, non-Hispanic	21 (3.4)	57 (7.1)	−3.7 (−6.1 to −1.3)	14 (6.5)	74 (8.8)	−2.3 (−6.4 to 1.8)
Education level						
High school or less	35 (5.7)	12 (1.5)	4.2 (2.0 to 6.3)	8 (3.7)	14 (1.7)	2.0 (−0.9 to 5.0)
Undergraduate or technical degree	418 (67.5)	425 (52.9)	14.6 (9.4 to 19.8)	146 (67.6)	434 (51.7)	15.9 (8.5 to 23.3)
Graduate or professional degree	164 (26.5)	365 (45.5)	−19.0 (−24.0 to −13.9)	60 (27.8)	392 (46.7)	−18.9 (−26.0 to −11.7)
Missing	2 (0.3)	1 (0.1)	0.2 (−0.5 to 0.9)	2 (0.9)	0	0.9 (−0.6 to 2.5)
Job classification						
MD	34 (5.5)	135 (16.8)	−11.3 (−14.6 to −8.0)	15 (6.9)	116 (13.8)	−6.9 (−11.3 to −2.5)
RN	233 (37.6)	253 (31.5)	6.1 (1.0 to 11.3)	78 (36.1)	229 (27.3)	8.8 (1.5 to 16.2)
Other	352 (56.9)	415 (51.7)	5.2 (−0.2 to 10.5)	123 (56.9)	495 (58.9)	−2.0 (−9.7 to 5.7)
Health insurance						
Private	554 (89.5)	761 (94.8)	−5.3 (−8.3 to −2.3)	196 (90.7)	803 (95.6)	−4.9 (−9.3 to −0.5)
Government	24 (3.9)	21 (2.6)	1.3 (−0.8 to 3.3)	8 (3.7)	16 (1.9)	1.8 (−1.2 to 4.8)
None	5 (0.8)	1 (0.1)	0.7 (−0.2 to 1.6)	0	0	…
Unknown	35 (5.7)	18 (2.2)	3.4 (1.2 to 5.6)	11 (5.1)	20 (2.4)	2.7 (−0.7 to 6.1)
Missing	1 (0.2)	2 (0.2)	−0.1 (−0.6 to 0.5)	1 (0.5)	1 (0.1)	0.3 (−0.9 to 1.6)
Presence of 2 or more comorbidities	310 (50.1)	404 (50.3)	−0.2 (−5.6 to 5.2)	125 (57.9)	474 (56.4)	1.4 (−6.2 to 9.1)

		Days, Mean (SD) Median (Q1–Q3)			Days, Mean (SD) Median (Q1–Q3)	
Time since most recent vaccination dose and earliest date of symptom onset	…	86 (38) 85 (61–106)	…	…	85 (36) 85 (62–104)	−0.1 (−0.7 to 0.3)

Statistical significance was defined as having a percentage point difference 95% confidence interval that does not include 0.

Abbreviation: SARS-CoV-2, severe acute respiratory syndrome coronavirus 2.

### Prevalence of Six-Week Symptoms

**Figure 2.**
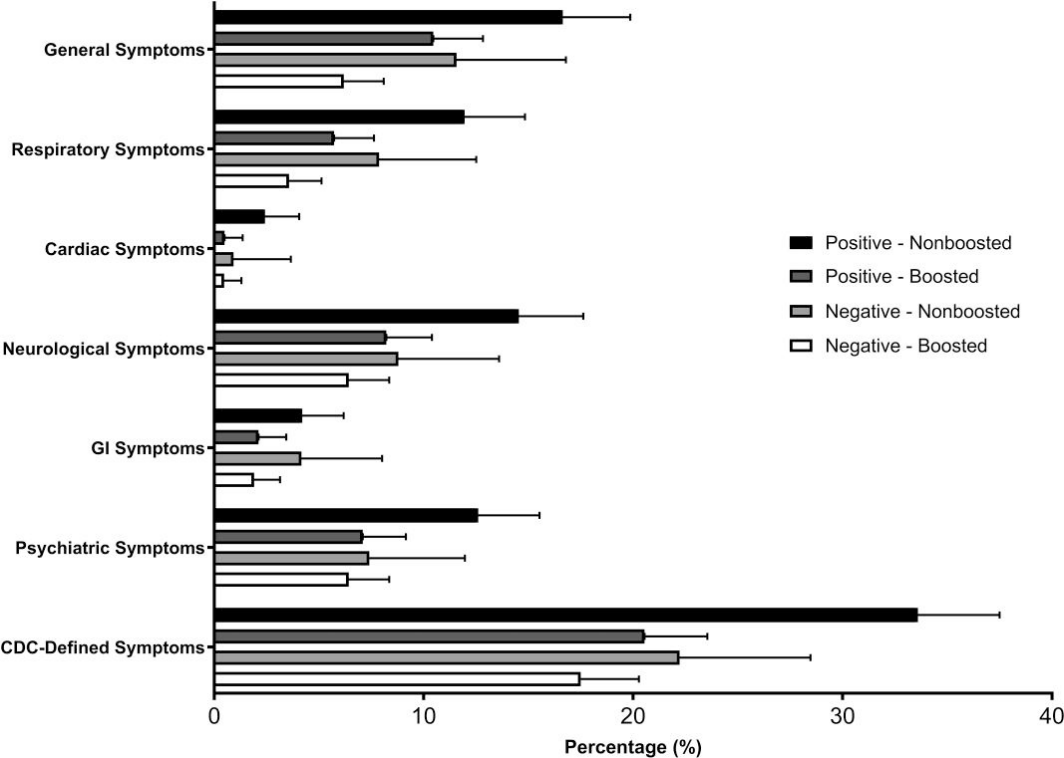
Frequency of symptoms at 6 weeks by SARS-CoV-2 infection and monovalent vaccine status. “Positive” and “negative” relate to the results of the SARS-CoV-2 test at the time of enrollment. “Nonboosted” means that participants had received 2 doses of an mRNA primary SARS-CoV-2 vaccination series, and “boosted” means that participants had additionally received an original monovalent mRNA SARS-CoV-2 vaccine dose. Error bars show the upper limit of the 95% CI. Prolonged symptoms include any symptom reported on the 6-week symptom survey; positive, SARS-CoV-2 positive; negative, SARS-CoV-2 negative; nonboosted, received 2 doses of mRNA vaccine as primary series; boosted, received 2 doses of mRNA vaccine plus an additional dose of original monovalent mRNA COVID-19 vaccine. Positive – HCP who had a positive SARS-CoV-2 test at enrollment. Negative – HCP who had a negative SARS-CoV-2 test at enrollment. Abbreviations: CDC, Centers for Disease Control and Prevention; COVID-19, coronavirus disease 2019; GI, gastrointestinal; HCP, health care personnel; SARS-CoV-2, severe acute respiratory syndrome coronavirus 2.

Among those with COVID-19, 373 (26.2%) reported symptoms at 6 weeks, with 156 (11.0%) reporting at least 1 neurologic symptom, 120 (8.4%) reporting at least 1 respiratory symptom, and 19 (1.3%) participants reporting at least 1 cardiac symptom. Among those who tested SARS-CoV-2-negative, 195 (18.5%) reported any symptoms at 6 weeks, 73 (6.9%) reported at least 1 neurologic symptom, 47 (4.5%) reported at least 1 respiratory symptom, and 6 (0.6%) participants reported at least 1 cardiac symptom (Supplementary Table 3). Compared with other causes of COVID-19-like symptoms, fatigue (11.2% vs 6.5%; difference, 4.6 PPs; 95% CI, 2.4–6.9 PPs) and sleep difficulty (6.5% vs 3.8%; difference, 2.8 PPs; 95% CI, 0.9–4.6 PPs) after 6 weeks were most strongly associated with having had COVID-19 (Figure 2).

### Association Between Six-Week Symptoms and Additional Vaccine Doses and Timing

Among participants who had COVID-19, those who had received an additional COVID-19 vaccine dose had a lower prevalence of any symptoms than those who had not (adjusted odds ratio [aOR], 0.55; 95% CI, 0.43–0.70). No statistical association was found between receipt of an additional vaccine dose and 6-week symptoms among those who were SARS-CoV-2-negative (aOR, 0.87; 95% CI, 0.59–1.29). A similar pattern was apparent for cardiac symptoms, neurologic symptoms, and psychiatric symptoms. Among participants with COVID-19, the strongest association between receipt of an additional vaccine dose and specific symptoms was for cardiac complaints (aOR, 0.25; 95% CI, 0.07–0.66) (Table 2). In a sensitivity analysis restricted to HCP who received their most recent vaccine dose within 16 weeks of their illness (n = 2182), we found similar results, consistent with our primary analysis. HCP who received an additional vaccine dose had lower odds of reporting symptoms, across all categories, at 6 weeks compared with those who received only the primary series (Supplementary Table 4).

**Table 2.** Unadjusted and Adjusted Odds of Reporting Symptoms at 6 Weeks by SARS-CoV-2 Infection and Vaccination Status During the Omicron Pandemic Period, December 2021 and April 2022

Model	SARS-CoV-2 Positive (n = 1422)		SARS-CoV-2 Negative (n = 1056)	
	Unadjusted Odds Ratio (95% CI)^a^	Adjusted Odds Ratio (95% CI)^b^	Unadjusted Odds Ratio (95% CI)^a^	Adjusted Odds Ratio (95% CI)^b^
General symptoms	0.51 (0.43 to 0.80)	0.64 (0.46 to 0.88)	0.50 (0.31 to 0.83)	0.63 (0.37 to 1.08)
Respiratory symptoms	0.45 (0.30 to 0.66)	0.46 (0.31 to 0.68)	0.43 (0.23 to 0.80)	0.43 (0.22 to 0.82)
Cardiac symptoms	0.20 (0.07 to 0.61)	0.25 (0.07 to 0.66)^c^	0.51 (0.09 to 2.81)	0.42 (0.09 to 2.45)^c^
Neurologic symptoms	0.53 (0.38 to 0.74)	0.58 (0.41 to 0.83)	0.71 (0.41 to 1.23)	0.86 (0.48 to 1.53)
GI symptoms	0.49 (0.27 to 0.92)	0.61 (0.32 to 1.12)^c^	0.45 (0.19 to 1.02)	0.60 (0.26 to 1.45)^c^
Psychiatric symptoms	0.53 (0.37 to 0.76)	0.59 (0.41 to 0.87)	0.86 (0.48 to 1.53)	1.06 (0.58 to 1.94)
Any symptoms	0.51 (0.40 to 0.65)	0.55 (0.43 to 0.70)	0.74 (0.51 to 1.07)	0.87 (0.59 to 1.29)

Statistical significance was defined as having an odds ratio 95% confidence interval that does not include 1.

Abbreviation: GI, gastrointestinal; SARS-CoV-2, severe acute respiratory syndrome coronavirus 2.

^a^Odds of reporting symptoms at 6 weeks among those with primary series plus an additional vaccine dose vs those vaccinated with primary series only.

^b^Odds of reporting symptoms at 6 weeks among those with primary series plus an additional vaccine dose vs those vaccinated with primary series after controlling for age, race and ethnicity, time to follow-up, job classification, influenza vaccine, and chronic medical conditions.

^c^Estimates come from a Firth regression due to sparsity issues.

We also examined the relationship between vaccine timing and symptom reporting at 6 weeks. The analysis showed that among HCP who had COVID-19, those who received their most recent vaccine dose (whether an additional dose or a primary series) within 16 weeks before their illness had lower odds of reporting respiratory symptoms (aOR, 0.69; 95% CI, 0.46–0.99), psychiatric symptoms (aOR, 0.68; 95% CI, 0.47–0.99), or any symptom (aOR, 0.68; 95% CI, 0.53–0.87) compared with those who received their last dose >16 weeks before illness. We did not observe any significant associations among COVID-19-negative HCP for this analysis (Supplementary Table 5).

In our analysis of only persistent symptoms (excluding new symptoms) in COVID-19-positive HCP, those who received an additional vaccine dose had lower odds of reporting persistent symptoms compared with those who received only the primary series (aOR, 0.48; 95% CI, 0.36–0.63). We did not find a similar association among those who were SARS-CoV-2-negative (aOR, 0.71; 95% CI, 0.45–1.12) (Supplementary Table 6).

## DISCUSSION

In this study, we found that symptoms were common among HCP 6 weeks after COVID-19 infection, with about a quarter of HCP reporting symptoms, and symptoms were ∼13% less prevalent in those who had received an additional vaccine dose during the Omicron variant–predominant period of the pandemic. We also found that a higher proportion of SARS-CoV-2-negative controls received an additional vaccine dose compared with the SARS-CoV-2-positive cases.

Previous research has suggested that vaccinated individuals experiencing COVID-19 have a lower risk of developing 6-week symptoms compared with those infected while unvaccinated [11, 25–27]. Many of these studies specifically examined the primary vaccine series rather than additional vaccine doses [11, 26, 28]. Building upon this body of evidence, our study extends these findings by demonstrating that additional vaccine doses may provide protection even among those diagnosed with COVID-19.

COVID-19 vaccination might lead to fewer 6-week symptoms among persons with symptomatic infection because of its effect in attenuating the severity of acute illness. Immunologic protection against severe disease is generally greater than that against milder infections, and receipt of an additional vaccine dose has been previously associated with attenuated case severity [29, 30]. Consistent with this observation, we found that receipt of an additional vaccine dose was associated with 23% lower prevalence of fever or cough at the time of enrollment, and severity of baseline symptoms was generally correlated with symptoms after 6 weeks.

In addition to the direct health benefits that vaccination has been demonstrated to provide (eg, reduction in symptom severity of acute illness or prolonged symptoms), vaccination has other benefits. In 1 study of 100 000 adults older than age 64 years, vaccination with an additional dose of COVID-19 vaccine following the initial 2 doses was a cost-effective strategy, providing a net monetary benefit of $3.4 million (benefit of $34 per person) [31]. Further, initial vaccine rollout averted ∼9 million symptomatic cases of COVID-19 and 700 000 hospitalizations, leading to an estimated $30.4 billion in direct health care cost savings and $43.7 billion in indirect cost savings [32].

Recently, the National Academies of Sciences, Engineering, and Medicine (NASEM) published a definition for long COVID: an infection-associated chronic condition that occurs after SARS-CoV-2 infection and is present for at least 3 months as a continuous, relapsing and remitting, or progressive disease state that affects 1 or more organ systems [33]. Multiple meta-analyses have demonstrated that COVID-19 vaccination reduces the likelihood of developing long COVID, with more vaccine doses often providing greater protection [12, 34]. Although our research focuses on symptoms reported at 6 weeks (not meeting the long COVID definition), it supports the existing literature suggesting that vaccination can reduce the odds of experiencing prolonged symptoms following acute illness. Future longer longitudinal prospective studies that include regular physician follow-up would provide a more robust design for evaluating the associations between long COVID, vaccination, and other risk factors.

We also used an analysis restricted to only our test-negative control group as a robustness check to confirm that the association between vaccination status and reduced 6-week symptoms is unlikely attributable solely to selection bias. We did not find statistical evidence that additional vaccine doses were associated with reduced symptoms in our SARS-Co-V-2-negative population, suggesting that the association between vaccination and 6-week COVID-19 symptoms is unlikely to be a spurious association caused by confounding or other systematic sources of bias in our study design [35, 36]. As no biologically plausible immune pathway exists for how COVID-19 vaccination might attenuate symptoms in non-COVID-19 participants, any observed relationship between vaccination and 6-week symptoms would likely be attributable to baseline differences in who chose to receive additional vaccine doses or the presence of residual confounding from unmeasured factors. Future studies measuring the effect of vaccination on delayed symptoms should include either interventional allocation designs (eg, randomization) or robust observational methods to account for selection bias in who chooses to be vaccinated. Research on delayed or prolonged symptoms would also benefit from objective or standardized symptom criteria diagnosed by a clinician to reduce the risk of bias due to participant selection or recall.

Several limitations should be acknowledged. First, HCP enrolled in the study may have initially tested negative for SARS-CoV-2 at enrollment but contracted unrecognized SARS-CoV-2 infection before completion of their 6-week follow-up. Second, we relied on self-reported symptoms, which introduces the potential for recall bias as people knew their SARS-CoV-2 status. Similarly, symptom severity was self-reported without specific severity benchmarks, which could introduce potential for misclassification bias and recall bias (as participants were not blinded). Third, the study's assessment of symptoms was conducted within a relatively short time frame; the longer-term effect of additional vaccinations remains uncertain. Fourth, our study focused on the use of the original monovalent vaccine product only; we were unable to assess associations for subsequent vaccine formulations. Fifth, our results could be partially attributable to “hybrid” immunity (from both prior SARS-CoV-2 infection and vaccination) among our vaccinated population. Sixth, we do not know whether symptoms were necessarily related to COVID-19 prolonged symptoms—some people with symptoms from other causes would have reported symptoms at our follow-up time. Seventh, our smaller sample size in the SARS-CoV-2-negative group leaves open the possibility that this portion of the analysis is underpowered. Lastly, as none of the participants in this analysis were hospitalized between enrollment and 6-week follow-up, the generalizability of our findings is limited to HCP who did not experience hospitalization or severe events during or following the acute phase of their COVID-19 illness.

In conclusion, we found that HCP who received an additional dose of COVID-19 vaccine had a lower prevalence of symptoms after 6 weeks than those who did not. We found that this effect was most pronounced in participants with COVID-19 (compared with those with negative SARS-CoV-2 tests), suggesting a specific and biologically plausible relationship. Our findings add to evidence of the benefit of COVID-19 vaccines, not only in preventing infection and severe illness, but also in improving recovery from COVID-19.

## Supplementary Material

ofae545_Supplementary_Data

## Supplementary Data

Supplementary materials are available at *Open Forum Infectious Diseases* online. Consisting of data provided by the authors to benefit the reader, the posted materials are not copyedited and are the sole responsibility of the authors, so questions or comments should be addressed to the corresponding author.
