# Supplementary material for: Symptoms Six Weeks After COVID-19 Are Reduced Among US Health Care Personnel Receiving Additional Vaccine Doses During the Omicron Period, December 2021–April 2022
Source: Open Forum Infect Dis. 2024 Sep 25;11(10):ofae545. doi: 10.1093/ofid/ofae545 (PMC11481461; doi:10.1093/ofid/ofae545)
Supplement: ofae545_Supplementary_Data [file ofae545_supplementary_data.pdf]

SUPPLEMENTAL MATERIALS

**Supplemental Tables**	**Page**
**Tables S1.** Symptom category definitions.	2
**Tables S2.** Prevalence of all baseline symptoms stratified by SARS-CoV-2 status.	3
**Table S3.** Prevalence of symptoms at 6 weeks by SARS-CoV-2 infection and monovalent vaccine status.	4
**Table S4.** Unadjusted and adjusted odds of reporting symptoms at 6-weeks by participants SARS-CoV-2 infection status and either being vaccinated with primary series only or primary series plus an additional vaccine dose among those who received an additional dose within 16 weeks of symptom onset, PREVENT (N=2182).	5
**Table S5.** Unadjusted and adjusted odds of reporting symptoms at 6 weeks by SARS-CoV-2 infection and vaccination timing to illness/testing during the Omicron Pandemic Period, December 2021 and April 2022.	6
**Table S6.** Unadjusted and adjusted odds of reporting persistent symptoms (symptoms at both enrollment and 6 weeks) by SARS-CoV-2 infection and vaccination status during the Omicron Pandemic Period, December 2021 and April 2022.	7

Supplemental Table S1. Symptom category definitions.

SYMPTOMS	Enrollment symptoms	General Symptoms (6 weeks)	Respiratory Symptoms (6 weeks)	Cardiac Symptoms (At 6 weeks)	Neurologic Symptoms (6 weeks)	GI Symptoms (6 weeks)	Psychiatric Symptoms (6 weeks)	Any Symptoms (6 weeks)
Abdominal Pain	X					X		X
Anxiety/Panic							X	X
Bruised Toes or Feet	X							
Chest Pain	X			X				X
Chills	X	X						X
Concentration Difficulty					X			X
Confusion					X			X
Cough	X		X					X
Depression							X	X
Diarrhea	X					X		X
Difficulty with Exercise		X						X
Dizziness					X			X
Fatigue	X	X						X
Fever	X	X						X
Headache	X				X			X
Joint Pains								X
Loss of appetite	X							
Loss of Taste/Smell	X				X			X
Memory Difficulty					X			X
Movement Problems					X			X
Myalgia	X							
Nausea	X					X		X
Rhinorrhea	X							
Rigors	X							
Shortness of Breath	X		X					X
Sinus or nasal congestion	X							
Severe Respiratory Illness	X		X					X
Sore Throat	X		X					X
Trouble Sleeping							X	X
Vomiting	X					X		X

Supplemental Table S2: Prevalence of all baseline symptoms stratified by SARS-CoV-2 infection status.

Baseline Symptoms	SARS-CoV-2 Positive N = 1422			SARS-CoV-2 Negative N = 1056		
	Prevalence			Prevalence		
	Primary Series Only N = 619	Primary Series Plus Additional Vaccine Dose N = 803	Percentage Point Difference (95% CI)	Primary Series Only N = 216	Primary Series Plus Additional Vaccine Dose N = 840	Percentage Point Difference (95% CI)
Shortness of breath or difficulty breathing	121 (19.5)	80 (10.0)	9.6 (5.7 to 13.5)	21 (9.7)	54 (6.4)	3.3 (-1.3 to 7.9)
Cough	448 (72.4)	471 (58.7)	13.7 (8.7 to 18.8)	102 (47.2)	330 (39.3)	7.9 (0.2 to 15.7)
Severe respiratory illness including pneumonia	8 (1.3)	3 (0.4)	0.9 (-0.2 to 2.0)	3 (1.4)	5 (0.6)	0.8 (-1.1 to 2.7)
Fever (greater than 100 F or 37.8 C)	275 (44.4)	171 (21.3)	23.1 (18.2 to 28.1)	31 (14.4)	91 (10.8)	3.5 (-1.9 to 8.9)
Fever OR cough	509 (82.2)	513 (63.9)	18.3 (13.7 to 23.0)	112 (51.9)	360 (42.9)	9.0 (1.2 to 16.7)
Myalgia (muscle aches)	284 (45.9)	276 (34.4)	11.5 (6.2 to 16.8)	40 (18.5)	150 (17.9)	0.7 (-5.4 to 6.7)
Changes in ability to smell or taste	138 (22.3)	89 (11.1)	11.2 (7.1 to 15.3)	15 (6.9)	30 (3.6)	3.4 (-0.5 to 7.3)
Chills	343 (55.4)	273 (34.0)	21.4 (16.2 to 26.7)	55 (25.5)	162 (19.3)	6.2 (-0.5 to 12.9)
Rigors (sudden feeling of cold with shaking)	96 (15.5)	59 (7.3)	8.2 (4.6 to 11.7)	7 (3.2)	30 (3.6)	-0.3 (-3.3 to 2.6)
Headache	461 (74.5)	470 (58.5)	15.9 (11.0 to 20.9)	133 (61.6)	438 (52.1)	9.4 (1.8 to 17.0)
Chest pain or chest tightness	99 (16.0)	75 (9.3)	6.7 (3.0 to 10.3)	16 (7.4)	44 (5.2)	2.2 (-1.9 to 6.3)
Sore throat	382 (61.7)	508 (63.3)	-1.6 (-6.8 to 3.7)	113 (52.3)	447 (53.2)	-0.9 (-8.7 to 6.9)
Rhinorrhea (runny nose)	283 (45.7)	369 (46.0)	-0.2 (-5.6 to 5.1)	93 (43.1)	342 (40.7)	2.3 (-5.3 to 10.0)
Sinus or nasal congestion	448 (72.4)	518 (64.5)	7.9 (2.9 to 12.8)	121 (56.0)	446 (53.1)	2.9 (-4.8 to 10.6)
Nausea or vomiting	136 (22.0)	92 (11.5)	10.5 (6.4 to 14.6)	34 (15.7)	109 (13.0)	2.8 (-2.9 to 8.4)
Diarrhea	156 (25.2)	104 (13.0)	12.3 (8.0 to 16.5)	25 (11.6)	111 (13.2)	-1.6 (-6.8 to 3.5)
Abdominal pain	39 (6.3)	15 (1.9)	4.4 (2.2 to 6.7)	13 (6.0)	44 (5.2)	0.8 (-3.0 to 4.6)
Loss of appetite	202 (32.6)	135 (16.8)	15.8 (11.2 to 20.5)	23 (10.6)	68 (8.1)	2.6 (-2.2 to 7.4)
Fatigue	467 (75.4)	506 (63.0)	12.4 (7.5 to 17.3)	115 (53.2)	389 (46.3)	6.9 (-0.8 to 14.7)
Bruised toes or feet	4 (0.6)	2 (0.2)	0.4 (-0.5 to 1.3)	0	1 (0.1)	-0.1 (-0.5 to 0.2)

Statistical significance was defined as having a percentage point difference 95% confidence interval that does not include zero.

Supplemental Table S3. Prevalence of symptoms at 6 weeks by SARS-CoV-2 infection and monovalent vaccine status.

	SARS-CoV-2 Positive (n = 1422)		SARS-CoV-2 Negative (n = 1056)	
**Model**	**Primary Series Only (n = 619) N (%)**	**Primary Series Plus Additional Vaccine Dose (n = 803) N (%)**	**Primary Series Only (n = 216) N (%)**	**Primary Series Plus Additional Vaccine Dose (n = 840) N (%)**
**General Symptoms**	103 (16.6)	84 (10.5)	25 (11.6)	52 (6.2)
**Respiratory Symptoms**	74 (12.0)	46 (5.7)	17 (7.9)	30 (3.6)
**Cardiac Symptoms**	15 (2.4)	4 (5.0)	2 (0.9)	4 (0.5)
**Neurologic Symptoms**	90 (14.5)	66 (8.2)	19 (8.8)	54 (6.4)
**GI Symptoms**	26 (4.2)	17 (2.1)	9 (4.2)	16 (1.9)
**Psychiatric Symptoms**	78 (12.6)	57 (7.1)	16 (7.4)	54 (6.4)
**Any Symptoms**	208 (33.6)	165 (20.5)	48 (22.2)	147 (17.5)

Supplemental Table S4. Unadjusted and adjusted odds of reporting symptoms at 6-weeks by participants SARS-CoV-2 infection status and either being vaccinated with primary series only or primary series plus an additional vaccine dose among those who received an additional dose within 16 weeks of symptom onset, PREVENT (N=2182).

	SARS-CoV-2 Positive (N = 1266)		SARS-CoV-2 Negative (N = 916)	
**Model**	**Unadjusted Odds Ratio (95% CI)** [1]	**Adjusted Odds Ratio (95% CI)** [2]	**Unadjusted Odds Ratio (95% CI)** [1]	**Adjusted Odds Ratio (95% CI)** [2]
**General Symptoms**	0.62 (0.45 to 0.85)	0.66 (0.47 to 0.93)	0.54 (0.32 to 0.90)	0.69 (0.40 to 1.20)
**Respiratory Symptoms**	0.50 (0.33 to 0.74)	0.50 (0.33 to 0.75)	0.45 (0.24 to 0.85)	0.43 (0.22 to 0.84)
**Cardiac Symptoms**	0.19 (0.05 to 0.65)	0.22 (0.06 to 0.65)[#]	0.61 (0.11 to 3.38)	0.51 (0.11 to 2.99)[#]
**Neurologic Symptoms**	0.57 (0.40 to 0.81)	0.63 (0.43 to 0.90)	0.78 (0.45 to 1.36)	0.93 (0.52 to 1.67)
**GI Symptoms**	0.47 (0.24 to 0.92)	0.58 (0.29 to 1.11)[#]	0.50 (0.22 to 1.17)	0.66 (0.28 to 1.63)[#]
**Psychiatric Symptoms**	0.56 (0.38 to 0.81)	0.61 (0.41 to 0.91)	0.94 (0.52 to 1.69)	1.14 (0.62 to 2.12)
**Any Symptoms**	0.54 (0.42 to 0.69)	0.56 (0.43 to 0.73)	0.78 (0.53 to 1.13)	0.92 (0.62 to 1.36)

[1]Odds of reporting symptoms at 6-weeks among those with the primary series plus an additional vaccine dose within 16 weeks of symptom onset versus those vaccinated with primary series only.

[2]Odds of reporting symptoms at 6-weeks among those with the primary series plus an additional vaccine dose within 16 weeks of symptom onset versus those vaccinated with primary series after controlling for age, race and ethnicity, time to follow-up, job classification, flu vaccine, and chronic medical conditions

#: Estimates come from a Firth Regression due to sparsity issues.

Statistical significance was defined as having an odds ratio 95% confidence interval that does not include one.

Supplemental Table S5. Unadjusted and adjusted odds of reporting symptoms at 6 weeks by SARS-CoV-2 infection and vaccination timing to illness/testing during the Omicron Pandemic Period, December 2021 and April 2022.

	SARS-CoV-2 Positive (N = 1422)		SARS-CoV-2 Negative (N = 1056)	
Model	Unadjusted Odds Ratio (95% CI) [1]	Adjusted Odds Ratio (95% CI) [2]	Unadjusted Odds Ratio (95% CI) [1]	Adjusted Odds Ratio (95% CI) [2]
General Symptoms	0.80 (0.59 to 1.09)	0.85 (0.61 to 1.16)	0.80 (0.49 to 1.30)	0.91 (0.55 to 1.51)
Respiratory Symptoms	0.69 (0.47 to 1.00)	0.67 (0.46 to 0.99)	0.54 (0.30 to 0.98)	0.54 (0.29 to 0.99)
Cardiac Symptoms	0.42 (0.16 to 1.12)	0.45 (0.16 to 1.12)[#]	2.08 (0.24 to 17.84)	1.49 (0.29 to 14.63)[#]
Neurologic Symptoms	0.71 (0.51 to 0.99)	0.75 (0.53 to 1.06)	0.89 (0.53 to 1.49)	0.94 (0.55 to 1.60)
GI Symptoms	0.49 (0.26 to 0.92)	0.56 (0.29 to 1.04)[#]	0.73 (0.32 to 1.67)	0.87 (0.37 to 2.04)
Psychiatric Symptoms	0.65 (0.45 to 0.93)	0.68 (0.47 to 0.99)	1.12 (0.65 to 1.93)	1.22 (0.70 to 2.13)
Any Symptoms	0.67 (0.53 to 0.85)	0.68 (0.53 to 0.87)	0.86 (0.62 to 1.21)	0.93 (0.65 to 1.31)

[1]Odds of reporting symptoms at 6-weeks among those who received a vaccine dose (regardless of primary series or additional dose) within 16 weeks of their illness/testing versus those who received a vaccine dose (regardless of primary series or additional dose) more than 16 weeks of their illness/testing.

[2]Odds of reporting symptoms at 6-weeks among those with a vaccine dose (regardless of primary series or additional dose) within 16 weeks of their illness/testing versus those with a vaccine dose (regardless of primary series or additional dose) more than 16 weeks of their illness/testing after controlling for age, race and ethnicity, time to follow-up, job classification, flu vaccine, and chronic medical conditions.

#: Estimates come from a Firth Regression due to sparsity issues.

Statistical significance was defined as having an odds ratio 95% confidence interval that does not include one.

Supplemental Table S6. Unadjusted and adjusted odds of reporting persistent symptoms (symptoms at both enrollment and 6 weeks) by SARS-CoV-2 infection and vaccination status during the Omicron Pandemic Period, December 2021 and April 2022.

	SARS-CoV-2 Positive (n = 1422)		SARS-CoV-2 Negative (n = 1056)	
**Model**	**Unadjusted Odds Ratio (95% CI)** [1]	**Adjusted Odds Ratio (95% CI)** [2]	**Unadjusted Odds Ratio (95% CI)** [1]	**Adjusted Odds Ratio (95% CI)** [2]
**Persistant Symptoms**	0.45 (0.34 to 0.59)	0.48 (0.36 to 0.63)	0.62 (0.40 to 0.96)	0.71 (0.45 to 1.12)

[1]Odds of reporting symptoms at 6-weeks among those with primary series plus an additional vaccine dose versus those vaccinated with primary series only.

[2]Odds of reporting symptoms at 6-weeks among those with primary series plus an additional vaccine dose versus those vaccinated with primary series after controlling for age, race and ethnicity, time to follow-up, job classification, influenza vaccine and chronic medical conditions

Statistical significance was defined as having an odds ratio 95% confidence interval that does not include one.
